# GP Trainees' perceptions and experiences of the training placement in Psychiatry - A qualitative study

**DOI:** 10.1192/bjo.2021.618

**Published:** 2021-06-18

**Authors:** Raja Adnan Ahmed, Rugiyya Saeed, Michal Tombs

**Affiliations:** 1 Aneurin Bevan University Health Board; 2 University Hospital of Wales; 3 Cardiff University

## Abstract

**Aims:**

This qualitative study aims to explore the leaning needs of the GP trainees for their psychiatry placements.

**Method:**

In this qualitative study, semi-structured interviews of eight former GP trainees were conducted. Data were transcribed and analysed using thematic analysis. Triangulation through multiple analysts” was used to improve the validity of the study

**Result:**

This study identified six key areas of learning needs for GP trainees during their psychiatry placement. i) the on-call experience which enabled the participants to learn how to manage acutely unwell patients in psychiatry, ii) learning the self-harm and suicidal risk assessment which is an important skill for a GP practising in primary care, iii) training in relation to psychiatric medication which enabled GPs to prescribe more confidently in the community, iv) exposure to the community psychiatry which was helpful in getting exposure to community-based clinical practice, v) learning from formal teaching activities which can be tailored to cover the relevant primary care related clinical topics and finally, vi) getting the opportunity to improve the communications skills during the psychiatry placement which is useful for all doctor in training.

**Conclusion:**

We recommend that detailed induction of the service setup is required before GP trainees start on-calls and a well-defined support network should be provided and explained to the training doctors. Risk assessment teaching should be delivered by formal training, regular supervision and discussions. Training on psychiatric medication especially in the context of GP prescribing should be considered as part of formal teaching experience. Opportunities to work with community mental health teams and outpatient clinics should be generated and offered to the trainees. Formal teaching sessions should be set up with an understanding of the GP training curriculum and their learning needs. Improvement of communications skills with exposure to difficult communication scenarios under supervision during psychiatric placement should be identified as an important area of learning for the GP trainees.

